# PNPLA3 148M/M Is More Susceptible to Palmitic Acid-Induced Endoplasmic Reticulum Stress-Associated Apoptosis in HepG2 Cells

**DOI:** 10.1155/2023/2872408

**Published:** 2023-02-14

**Authors:** Yunzhi Chen, Xuemei Yan, Tian Wang, Hongrong Deng, Xiaojie Deng, Fen Xu, Hua Liang

**Affiliations:** ^1^Department of Endocrinology and Metabolism, Third Affiliated Hospital of Sun Yat-Sen University, Guangdong Provincial Key Laboratory of Diabetology, Guangzhou 510630, China; ^2^Guangdong Provincial People's Hospital, Guangzhou 510080, China; ^3^Department of Endocrinology and Metabolism, Joint Service Support Force 903 Hospital, Hangzhou 310005, China

## Abstract

**Background:**

Patatin-like phospholipase domain-containing 3 (PNPLA3) is a major susceptibility gene for nonalcoholic fatty liver disease (NAFLD), and its rs738409 (I148M) polymorphism is associated with the occurrence and progression of NAFLD. Endoplasmic reticulum (ER) stress-related hepatocyte lipoapoptosis contributes to the progress of NAFLD. PNPLA3 is also known as a member of the calcium-independent phospholipase A2*ε* family, which can hydrolyze fatty acids to generate lysophosphatidylcholine (LPC) that induces ER stress-related hepatocyte lipoapoptosis. Whether the PNPLA3 risk genotype 148M/M is involved in more severe ER stress-associated lipoapoptosis is unclear.

**Methods:**

A PNPLA3148I knock-in HepG2 cell model was constructed based on HepG2 expressing PNPLA3 148M/M using the Cas9/sgRNA system. PNPLA3 148M/M, I/M, and I/I cells were treated with 0.3 mM palmitic acid (PA) for 24 h to induce lipid deposition. Cellular lipid deposition was detected by oil red staining. Apoptosis was observed by TUNEL. LPC was determined by ELISA, and the expression of PNPLA3, the ER stress marker Bip, molecules involved in the ER stress PERK/elF-2a pathway, and its downstream C/EBP homologous protein (CHOP)-mediated apoptotic pathway were detected by western blot.

**Results:**

The results showed no difference in PNPLA3 basal expression and basal hepatocyte lipid content between the three genotypes of cells. Lipid deposition and apoptosis were more severe in PNPLA3 148M/M and 148I/M cells than in I/I cells after PA treatment. PA-induced upregulation of protein expression of Bip, ER stress-responsive PERK pathway molecules p-PERK, p-eIF2*α*, CHOP, and CHOP-associated apoptotic molecules PUMA and Bax were more pronounced in PNPLA3 148M/M cells than in PNPLA3 148I/I cells. The basal LPC levels and the PA-treated increase of LPC levels in the cell culture supernatants did not differ between the three genotypic cells.

**Conclusion:**

PNPLA3 148M/M cells were more susceptible to PA-induced lipid deposition and ER stress-related apoptosis than 148I/I cells, and the proapoptotic susceptibility of PNPLA3 148M/M is independent of LPC.

## 1. Introduction

With changes in lifestyle and dietary habits, the prevalence of nonalcoholic fatty liver disease (NAFLD) has increased significantly compared to the past decade, with a prevalence of 20% in urban areas of China [1]. NAFLD refers to excessive hepatic fat deposition, excluding obvious liver damage factors, and includes the entire disease spectrum of simple steatosis, steatohepatitis (NASH), and cirrhosis.

Patatin-like phospholipase structural domain 3 (PNPLA3) is the major susceptibility gene for NAFLD, and its PNPLA3 I148M (rs738409, C > G) is strongly associated not only with hepatic fat content [2] but also with the progression of NAFLD to NASH, fibrosis, and cirrhosis [3]. These aforementioned associations have also been replicated in different geographical regions and ethnic populations [4]. The frequency of the G/G genotype (148M/M) is 14.1% in East Asian populations based on genome project data. PNPLA3 I148M was also found to be significantly associated with NALFD susceptibility and with the histologic severity of NAFLD in the Chinese and Japanese populations [5–8]. The mechanism by which PNPLA3 I148M promotes NAFLD progression is unclear. Hepatocyte lipoapoptosis plays an important role in the progression of NAFLD [9]. Activation of the mitochondrial cascade response and related CHOP signaling by endoplasmic reticulum (ER) stress is thought to be a key mechanism of hepatocyte apoptosis [10, 11]. Unlike PNPLA3 I/I, which is subcellularly distributed in the cytosol, the distribution of PNPLA3 148M/M is mainly concentrated in lipid droplets. Furthermore, our previous study found that overexpression of PNPLA3 148M/M rather than 148I/I activated ER stress [12]. Therefore, we speculated that hepatocytes carrying mutant PNPLA3 148M/M may respond more severely to high-fat-induced lipoapoptosis than those cells carrying wild-type PNPLA3 148I.

In addition, PNPLA3 has a protein sequence homologous to the noncalcium-dependent phospholipase A2 (iPLA2) family ((G/A) XGXXG) and GXSXG), as well as a structural domain homologous to patatin [13]. In vitro studies have shown that recombinant PNPLA3 possesses phospholipase activity [13], indicating its ability to hydrolyze sn-2 fatty acids from membrane phospholipids to generate lysophospholipid [14], primarily lysophosphatidylcholine (LPC) [15], which has proapoptotic effects [16]. In the hepatocyte model of palmitic acid (PA)-induced lipid deposition, iPLA2 was found to be involved in the mechanism of hepatocyte lipoapoptosis by increasing LPC content and then activating ER stress-related CHOP signaling [16]. Whether PA-induced LPC production differs in human hepatocytes carrying wild-type and mutant genotypes of PNPLA3 I148M, subsequently leading to inconsistent ER stress-CHOP-related apoptotic responses, has not been reported.

Therefore, the aim of this study was to compare the differences in free fatty acid-induced lipid deposition, LPC production, apoptosis, ER stress, and its related CHOP pathway activation among HepG2 cells expressing PNPLA3 148I/I, I/M, and M/M. Palmitic acid, the most abundant saturated fatty acid in the human body, was used in this study to induce a cellular model of NAFLD in vitro because of its apparent lipotoxicity involved in the pathogenesis of NAFLD [17].

The study will provide new ideas to reveal the mechanisms by which PNPLA3 I148M promotes NAFLD progression.

## 2. Materials and Methods

### 2.1. Constructing PNPLA3 148I/I Knock-In HepG2 Cells by Cas9/sgRNA

PNPLA3 148 I/I and 148 I/M HepG2 cell lines were generated using original HepG2 carrying the PNPLA3 148M/M genotype as a template by CRISPR/Cas9 based on the extreme genome editing (EGE) system as described in Figure S1. The three designed SgRNAs were loaded into the precut pCS linear plasmid with an existing Caspase9 expression element by sticky end ligation reaction using BbsI restriction endonuclease as described previously [18]. The pCS vector was cotransfected with the precut pUCA luciferase reporter vector containing the shear target fragment for 24 hours in HEK293T cells, and the sgRNA (up: CACCGCTTCATGCCTTTCTACAG; down: AAACCTGTAGAAAGGCATGAAGC) with the strongest luciferase activity, i.e., the strongest on-target activity, was selected for subsequent experiments. The left homologous arm containing PNPLA3 148I (c. 444C), the right homologous arm, and the resistance gene PuroR were ligated into the TV-4G vector by the AIOTM Cloning Kit to form the donor vector. The pCS vector and the donor vector are cotransfected with HepG2 cells; the PAM on the target DNA is recognized by SgRNA and sheared by caspase9, and the DNA is homologously repaired by the homologous template with 148I, changing the base sequence encoding the amino acid at position 148 from ATG to ATC. The transfected cells were then screened for positive clones and identified by sequencing.

### 2.2. Cell Culture and Treatment

HepG2 cells were cultivated under standard conditions containing Dulbecco's Modified Eagle's Medium (DMEM) supplemented with 10% fetal bovine serum (FBS, Gibco BRL, USA) at 37°C with 5% CO_2_. A 5 mM Palmitate (PA, #P9767; Sigma-Aldrich):10% BSA (4 : 1 molar ratio) stock solution was prepared by adding 50 ml of 100 mM PA to 950 ml of 10% FFA-free BSA solution and then diluted to 0.3 mM final. Cells were treated with 0.3 mM PA for 24 hours to induce hepatic steatosis. Cells treated with FFA-free BSA were prepared as control.

### 2.3. Oil Red O Staining and Triglyceride Assay

Lipid deposition in HepG2 cells was observed by Oil Red O (Sigma-Aldrich) staining. Briefly, cells were fixed with formaldehyde, stained with Oil Red O working solution for 30 min, and the nuclei were counterstained with hematoxylin for 1 min. Cells were imaged using a Leica DMi1 I inverted microscope (Leica, Wetzlar, Germany) at 400x magnification. The Oil Red O dye was extracted from the stained cells using 100% isopropanol. Quantification of lipid was performed by measuring the optical density (OD) reading of Oil Red O at a wavelength of 510 nm using a spectrophotometer. Intracellular triglycerol (TG) content was detected using a TG assay kit (Biovison, Milpitas, CA) according to the kit instructions.

### 2.4. TUNEL Assay

After cells were digested by trypsin, equal amounts of cells were taken from each group and incubated for 24 hours in 24-well plates with cover glass-slides and then incubated with 0.3 mM PA for 24 hours. The slides were removed and washed twice with PBS, and the cells were fixed with formaldehyde. Apoptotic cells were stained according to the TUNEL assay instructions; hematoxylin stained nuclei for several seconds, dehydrated in gradient alcohol, air dried, and sealed with neutral gum. Images were acquired by microscopy. Apoptosis rate (%) = positive TUNEL staining/total nuclei × 100%.

### 2.5. Lysophosphatidylcholine Assay

The supernatants of PA-treated cells and their control cells were collected, centrifuged to remove cellular sediment, and then operated according to the instructions of the human lysophosphatidylcholine (LPC) ELISA kit (Cloud-Clone Corp., Cat no. CEK621Ge).

### 2.6. Western Blot

Cells were washed twice with precooled PBS, and then the total cellular protein was extracted by adding RIPA lysate containing a protease inhibitor, and protein concentration was determined by the BCA method. A volume of 10 ul (4 ug/ul) protein was taken for gel electrophoresis, and the protein was transferred to the PVDF membrane. After being closed with 5% skimmed milk for 1 hour at room temperature, the primary antibody was incubated overnight, including anti-PNPLA3 (ab81874; Abcam), anticleaved caspase3 (#9664, Cell Signaling Technology), anti-BIP (#3183, Cell Signaling Technology), anti-PERK (#5683, Cell Signaling Technology), anti-p-PERK (Thr981) (sc-32577, Santa Cruz), anti-eIF-2a (#9722, Cell Signaling Technology), anti-p-eIF-2a (Ser51) (#9721, Cell Signaling Technology), anti-CHOP (#2895, Cell Signaling Technology), anti-PUMA (#4976, Cell Signaling Technology), anti-BAX (#5023, Cell Signaling Technology), and anti-*β*-actin (#4970, Cell Signaling Technology). The membrane was then washed with TBST and incubated with fluorescently labeled secondary antibodies for 1 hour at room temperature. Protein bands were visualized with an infrared fluorescence scanning imaging system (Odyssey ® DLx Imaging System, LI-COR Biosciences). Each experiment was repeated a minimum of three times.

### 2.7. Statistical Analysis

Statistical analysis was performed using SPSS 13.0. Normally distributed measures were shown as the mean ± standard error. Fisher's least significant difference (LSD) method was used for pairwise multiple comparison. Differences were considered statistically significant at *P* < 0.05.

## 3. Results

### 3.1. PA-Induced Lipid Deposition Was Greater in PNPLA3148I/M and M/M Cells than in 148I/I Cells

**Figure 1:**
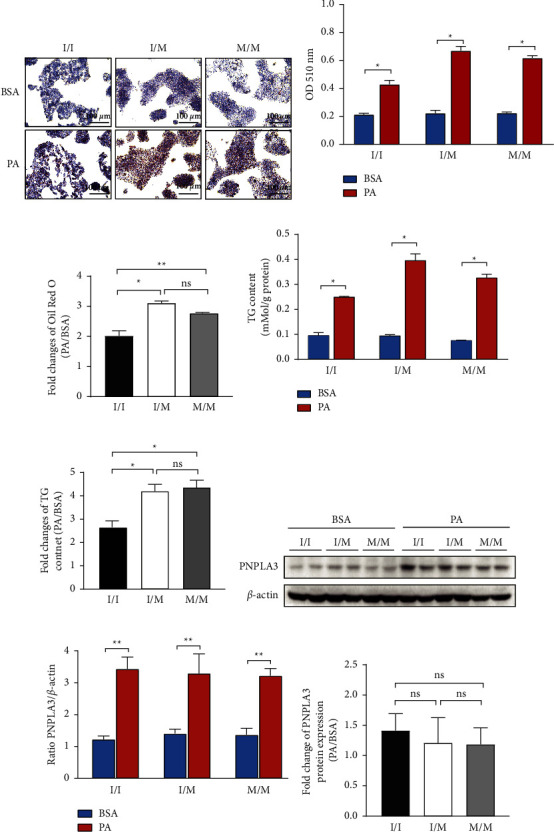
Lipid deposition and PNPLA3 expression. The three genotypic HepG2 cells were treated with 0.3 mM PA for 24 h. (a) Visualized oil red O staining using a research-grade inverted microscope at 400x magnification. The scale bar in each image represents 100 *µ*m. (b) Quantification of lipid measured by elution of oil red-O staining. (c) Fold change (PA/BSA) of (b). (d) Quantification of the intracellular TG content. (e) Fold change (PA/BSA) of (d). (f) The protein level of PNPLA3 detected by western blot. (g) The quantitative analysis of gray value based (f) performed using image-pro plus software. (h) Fold change (PA/BSA) of (g). Biological replicates (*N* = 3) were performed per group. Quantitative data are presented as the mean ± SD (*N* = 3 independent experiments). ^*∗*^*P* < 0.05; ^*∗∗*^*P* < 0.01.

Oil Red O staining showed that lipid deposition in the three genotypic HepG2 cells was not significantly different at the baseline and significantly increased after PA treatment (Figures 1(a) and 1(b)). The extent of PA-induced lipid deposition differed among the three genotypic cells, i.e., the increment of PA-induced lipid deposition in PNPLA148M/M and I/M cells was significantly greater than that in I/I cells (Figure 1(c)). The results of intrahepatocellular TG content measurements at the baseline and on PA treatment in the three genotypic cells were consistent with the results of Oil Red O staining (Figure 1(d)). The increase in TG content was significantly higher in PA-induced PNPLA148 I/M and M/M cells than in I/I cells (fold change in TG content before and after PA treatment in I/I, I/M, and M/M cells: 2.63-, 4.19-, and 4.35-fold, respectively) (Figure 1(e)). To understand whether the different responses of HepG2 cells among the three genotypic groups to PA-induced lipid deposition were due to differences in PNPLA3 expression, we determined the protein expression of PNPLA3. The results showed that PNPLA3 protein expression in the three genotypic cells was comparable at the baseline (Figures 1(f) and 1(g)). PA treatment resulted in a significant upregulation of PNPLA3 expression in each genotypic cell (Figures 1(f) and 1(g)), and the upregulated fold change was not statistically different among the three genotypic cells (Figure 1(h)).

### 3.2. PA Induced More Apoptosis in PNPLA3148I/M and M/M Cells than in 148I/I Cells

**Figure 2:**
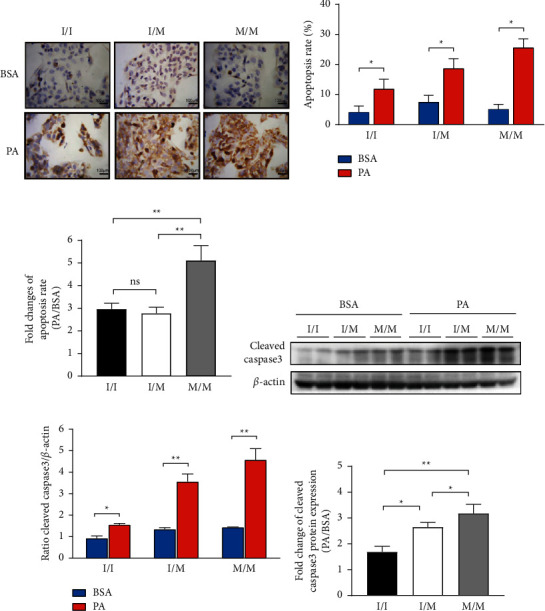
Apoptosis in HepG2 cells and cleaved-caspase-3 expressing. The three genotypic HepG2 cells were treated with 0.3 mM PA for 24 h. (a) The visualized TUNEL staining assay using a research-grade inverted microscope at 400x magnification. The scale bar in each image represents 100 *µ*m. (b) The percentage of TUNEL-positive cells. (c) Fold change (PA/BSA) of (b). (d) The protein level of cleaved-caspase-3 detected by western blot. (e) The quantitative analysis of gray value based (d) performed using image-pro plus software. (f) Fold change (PA/BSA) of (e). Biological replicates (*N* = 3) were performed per group. Quantitative data are presented as the mean ± SD (*N* = 3 independent experiments). ^*∗*^*P* < 0.05; ^*∗∗*^*P* < 0.01.

TUNEL analysis showed no significant difference in baseline apoptosis levels in cells of the three genotypes (Figures 2(a) and 2(b)). Compared to BSA control, PA significantly increased the level of apoptosis in each genotypic cell (Figures 2(a) and 2(b)), with a 2.96-fold, 2.78-fold, and 5.12-fold increase in the apoptosis rate in PNPLA3 148I/I, I/M, and M/M cells, respectively (Figure 2(c)), indicating that the apoptotic response of PNPLA3 148M/M cells induced by PA was significantly higher than that of 148I/I cells. Consistent with the TUNEL results, activated apoptosis executive protein caspase-3 (cleaved caspase-3) protein expression was not significantly different at the baseline and was significantly upregulated after PA treatment (Figures 2(d) and 2(e)), and the PA-induced increment of cleaved caspase-3 expression was significantly higher in 148I/M and M/M cells than that in I/I cells (Figure 2(f)).

### 3.3. PA Induced Greater Activation of the ER Stress-Responsive PERK Pathway and Its Downstream CHOP-Mediated Apoptosis Pathway in PNPLA3148I/M and M/M Cells than in 148I/I Cells

**Figure 3:**
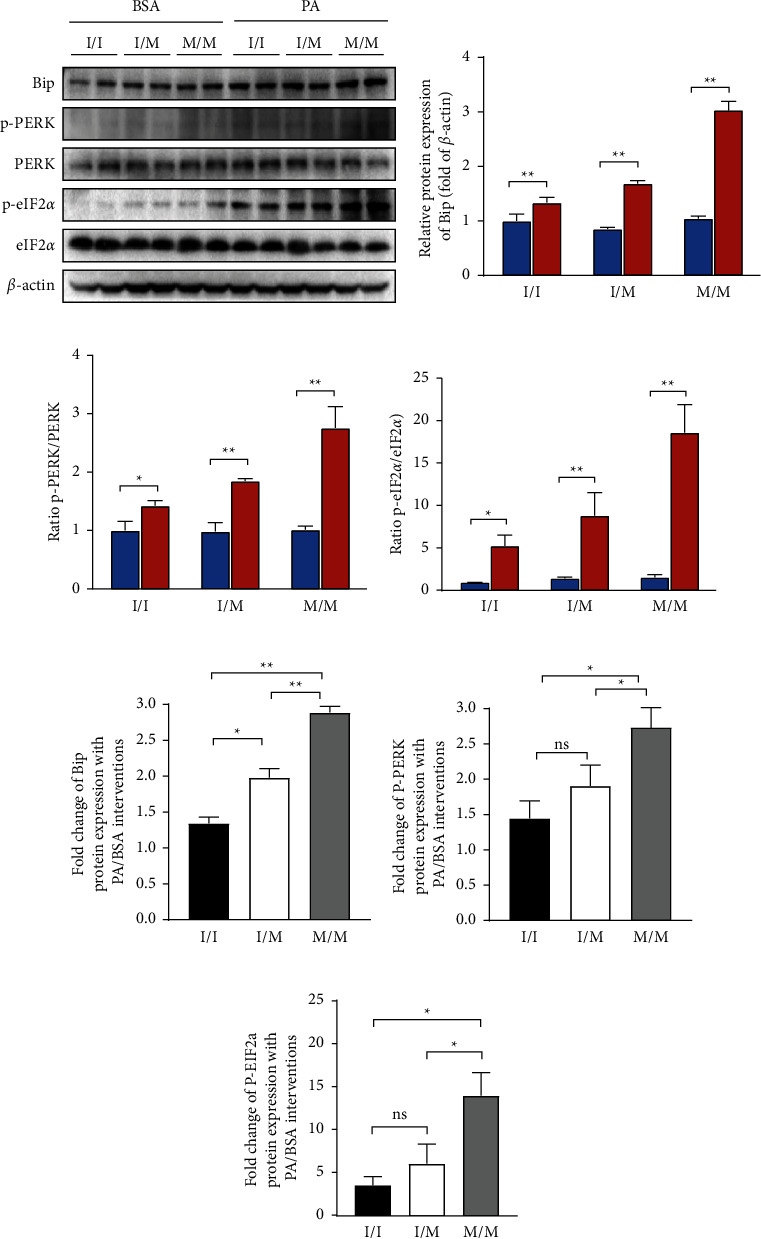
The protein expression of molecules involved in the ER stress responsive PERK pathway. (a) The protein level of Bip and total and phosphorylation levels of PERK and eIF-2a protein detected by western blot. (b–d) The quantitative analysis of gray value based (a) performed using image-pro plus software. (e–g) Fold change (PA/BSA) of (b–d). Biological replicates (*N* = 3) were performed per group. Quantitative data are presented as the mean ± SD (*N* = 3 independent experiments). ^*∗*^*P* < 0.05; ^*∗∗*^*P* < 0.01.

**Figure 4:**
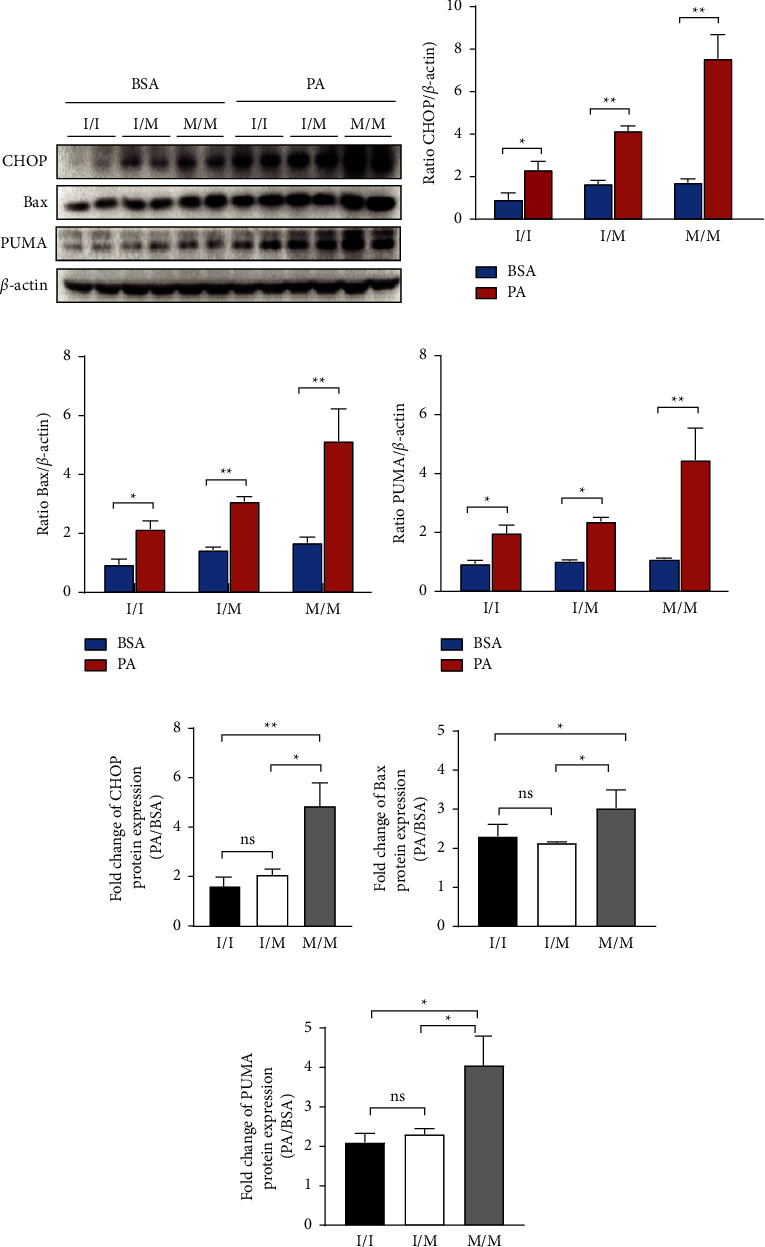
The protein expression of molecules involved in the CHOP apoptotic pathway. (a) The protein level of Bip, bax, and PUMA detected by western blot. (b–d) The quantitative analysis of gray value based (a) performed using image-pro plus software. (e–g) Fold change (PA/BSA) of (b–d). Biological replicates (*N* = 3) were performed per group. Quantitative data are presented as the mean ± SD (*N* = 3 independent experiments). ^*∗*^*P* < 0.05; ^*∗∗*^*P* < 0.01.

Protein expression in the PA-induced ER stress-responsive PERK signaling pathway and its downstream CHOP-mediated apoptotic pathway was examined using western blotting. As shown in Figures 3(a) and 3(b), the expression of the ER stress marker Bip was not significantly different between the three genotypic cells at the baseline and was significantly increased after PA treatment. Total protein and phosphorylated protein expression of PERK pathway molecules, PERK and eIF-2a, are comparable at the baseline. In PA-treated each genotypic cells, total PERK and eIF-2a expression did not change significantly compared with BSA control, but phosphorylated PERK and eIF-2a expression were markedly upregulated (Figure 3(a)), which resulted in an increase in the phosphorylated/total protein ratio of PERK and eIF-2a (Figures 3(c) and 3(d)). The previous PA-induced increase in protein expression was significantly higher in 148M/M cells than in I/M and I/I cells (Figures 3(e)–3(g)). Similarly, the expression of CHOP apoptotic pathway molecules, CHOP, PUMA, and Bax, was similar at the baseline and significantly increased after PA treatment in the three genotypic cells, as shown in Figures 4(a)–4(d). The aforementioned PA-induced magnitude of the increase in protein expression was most pronounced in 148M/M cells (Figures 4(e)–4(g)).

### 3.4. No Difference in LPC Levels in the Culture Cell Supernatant of PNPLA3 148 I/I, IM, and M/M HepG2 Cells at the Baseline and after PA Treatment

**Figure 5:**
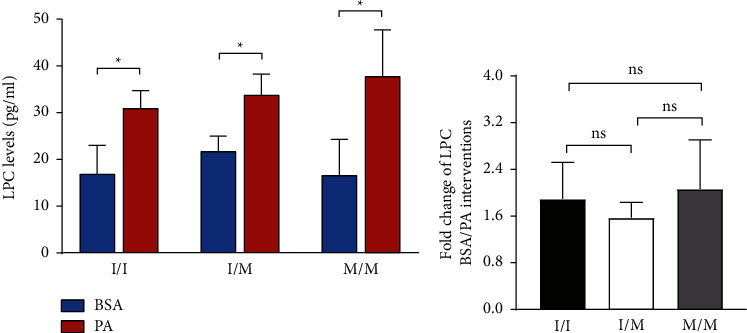
LPC levels of the culture cell supernatant. (a) LPC levels of the culture cell supernatant detected by ELISA. (b) Fold change (PA/BSA of (a). Biological replicates (*N* = 3) were performed per group. Quantitative data are presented as the mean ± SD (*N* = 3 independent experiments). ^*∗*^*P* < 0.05.

LPC is a secreted substance, and detection of LPC in the cell supernatant reflects its intracellular level. Therefore, we measured the LPC content in the supernatant of three genotypic cells under PA-treated and untreated conditions using ELISA. The results are shown in Figure 5. There was no significant difference in LPC levels between the three genotypic cells without PA treatment (Figure 5(a)). After PA treatment, the supernatant LPC levels increased in all three genotypic cells (Figure 5(a)), and the PA-induced LPC increase did not differ among the three genotypic cells (fold changes of 1.89, 1.57, and 2.06 for I/I, I/M, and M/M cells, respectively) (Figure 5(b)).

## 4. Discussion

In the present study, we compared for the first time the differences in responsiveness to PA-induced lipid deposition and lipoapoptosis in three genotypic HepG2 cells of PNPAL3 I148M and the possible mechanisms. Our results showed that PA-induced increases in lipid deposition and apoptosis were greater in 148M/M cells compared with 148I/I cells, and the underlying mechanism may involve a more pronounced response of PNPLA 148M/M cells to PA-induced activation of the ER stress PERK pathway and its related CHOP signaling.

Hepatocyte apoptosis plays an important role in promoting NAFLD progression [19]. Activation of ER stress is involved in saturated free fatty acid palmitate-induced hepatocyte lipoapoptosis [20]. CHOP is a proapoptotic transcription factor. Three major ER stress-related response pathways, PERK, ATF6, and IRE-1, all activate CHOP, with the PERK pathway being the most important [21]. The mechanism of LPC-induced lipoapoptosis is largely similar to that of PA-associated apoptosis [22]. PA-induced lipoapoptosis in L02 and HepG2 cells is achieved through the PERK/ATF4/CHOP signaling pathway [23], and LPC induces apoptosis through activation of eIF2*α* and CHOP in huh-7 cells [22]. Therefore, this study focused on the PERK pathway. PUMA is known as an important endogenous apoptotic molecule that is transcriptionally regulated by CHOP and interacts with proapoptotic proteins such as BAX to deliver mitochondrial apoptotic signals [11]. In the present study, we found that PA-induced apoptosis, activation of ER stress-responsivePERK-eIF2 signaling, and upregulation of its downstream expression of CHOP apoptotic pathway molecules were more pronounced in M/M cells than in I/I cells. This suggests that the higher activation of ER stress-responsive PERK-eIF2-CHOP signaling in M/M cells than in I/I cells may be one of the mechanisms by which PNPLA3 148 M/M is more susceptible to NAFLD progression.

PNPLA3, also known as iPLA2*ε* [13], has a mild phospholipase activity *in vitro* and theoretically catalyzes the production of proapoptotic LPC from membrane phospholipids. Since the mechanism of LPC-induced hepatocyte lipoapoptosis may involve activation of the ER stress-responsive PERK-eIF2a signaling pathway to promote CHOP expression [22], therefore, in order to understand whether the more significant apoptotic response of M/M cells than I/I cells to ER stress is attributable to differences in PA-induced LPC production, we compared LPC contents in the supernatants of PA-treated HepG2 cells carrying different PNPLA3 I148M genotypes. We found no significant differences among the three genotypic cells in terms of baseline LPC levels and PA-induced fold increases in LPC levels. This result suggests that the more pronounced ER stress and associated apoptotic responses in PNPLA3 148M/M cells are independent of the role of LPC. Our results are consistent with previous studies that found no different LPC levels between mice overexpressing liver-specifically human PNPLA3 148M/M and wild-type 148I/I [24]. In addition, no correlation was found between PNPLA3 I148M and serum LPC levels in NAFLD patients in a study on the effect of PNPLA3 single nucleotide polymorphisms on serum lipidomics in NAFLD patients [25]. Thus, we and other studies illustrate that PNPLA3 I148M is involved in the development and progression of NAFLD independent of its phospholipase activity.

PNPLA3 I148M was mainly localized to lipid droplets (LDs) [26, 27], which is the basis of PNPLA3 148M/M-associated hepatic steatosis. Therefore, the more pronounced ER stress activation and lipoapoptosis seen in 148M/M cells than 148I/I cells in the present study may be attributed to higher accumulation on LDs and more pronounced hepatocyte steatosis in PNPLA3 148M/M. Furthermore, our previous study showed that overexpression of PNPLA3 148M/M, but not PNPLA3I/I, in HepG2 activated the IRE-1a/JNK/c-Jun pathway of the ER stress response and promoted inflammation [12]. Therefore, it cannot be excluded that PNPLA3 148M/M itself may be involved in the activation of ER stress. The mechanism by which PNPLA3 148M/M promotes ER stress apoptosis requires subsequent in-depth study.

In summary, this study found that PNPLA3 148I/M and M/M cells were more susceptible to PA-induced lipid deposition and ER stress-related apoptosis compared with 148I/I cells. The proapoptotic susceptibility of PNPLA3 148M/M was independent of LPC. The study provides new insight into the mechanism by which PNPLA3 I148M promotes NAFLD progression.

## Data Availability

The data that support the findings of this study are available from the corresponding author upon reasonable request.

## Supplementary Materials

Supplementary Materials The supplemental figure depicts the construction of the PNPLA3 148I/I knock-in HepG2 cell by CRISPR/Cas9 based on the extreme genome editing (EGE) system. Knock-in HepG2 cells harbored a point mutation in the human PNPLA3 gene to substitute the p.148M with 148I, which corresponded to a change from c.444G to C. Three sgRNAs were designed by targeting p.148M and screened for on-target activity using a Universal CRISPR Activity Assay. Sanger sequencing of DNA from homozygous knock-in (PNPLA3 I148I) cell lines and heterozygous knock-in (PNPLA3 I148M) cell lines is shown in Figure S1. Black rectangle: c.444C, single peak and red rectangle: c.444 C/G, mixed peaks.
