# Supplementary figures and images for: PNPLA3 148M/M Is More Susceptible to Palmitic Acid-Induced Endoplasmic Reticulum Stress-Associated Apoptosis in HepG2 Cells
Source: Int J Endocrinol. 2023 Feb 14;2023:2872408. doi: 10.1155/2023/2872408 (PMC9943609; doi:10.1155/2023/2872408)

### PNPLA3 I148I knock-in cell lines

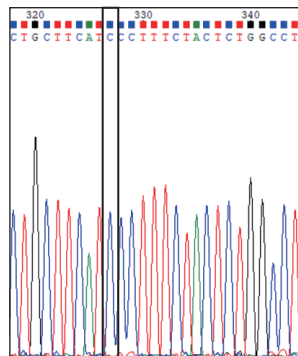

clone No.

clone1

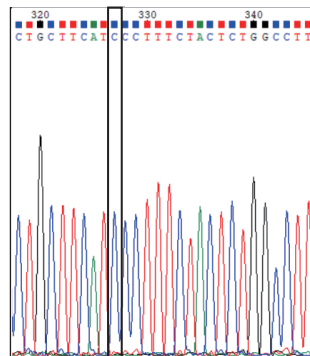

clone25

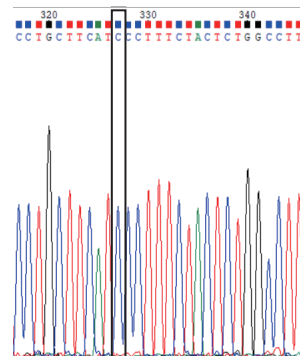

clone26

### PNPLA3 I148M knock-in cell line

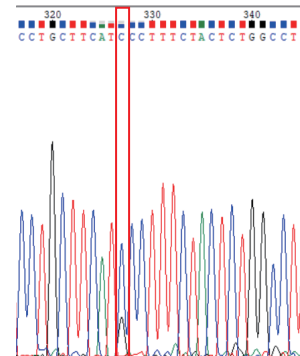

clone20

Supplement: Supplementary Materials — The supplemental figure depicts the construction of the PNPLA3 148I/I knock-in HepG2 cell by CRISPR/Cas9 based on the extreme genome editing (EGE) system. Knock-in HepG2 cells harbored a point mutation in the human PNPLA3 gene to substitute the p.148M with 148I, which corresponded to a change from c.444G to C. Three sgRNAs were designed by targeting p.148M and screened for on-target activity using a Universal CRISPR Activity Assay. Sanger sequencing of DNA from homozygous knock-in (PNPLA3 I148I) cell lines and heterozygous knock-in (PNPLA3 I148M) cell lines is shown in Figure S1. Black rectangle: c.444C, single peak and red rectangle: c.444 C/G, mixed peaks. [file 2872408.f1.pdf]
